# Why We Still Need Reliable Animal Models

**DOI:** 10.3390/pathophysiology27010006

**Published:** 2020-12-14

**Authors:** Olga Pechanova

**Affiliations:** Institute of Normal and Pathological Physiology, Centre of Experimental Medicine, Slovak Academy of Sciences, 813 71 Bratislava, Slovakia; olga.pechanova@savba.sk

Animal models are still an essential tool for identifying key molecular mechanisms and pathophysiological manifestations of different diseases, as well as for the analysis of the most effective intervention for the treatment and reduction of the consequences of pathophysiological conditions. Their use is profoundly important for a thorough demonstration of the effectiveness and safety of drugs and obtaining basic information on the route of administration, pharmacokinetics, and pharmacodynamics. However, each model system has different specific limitations and disadvantages, which may be the reason why no animal model has fully reproduced all the key features of the severe form of COVID-19 yet. This deficit in preclinical modelling is very serious, as vaccines are not yet widely available and their use will be accompanied by several limitations.

Therefore, there is an urgent need to identify potential treatment for patients who have already experienced SARS-CoV-2 infection and are at risk of progressing to severe COVID-19. Significant evidence of the effectiveness of therapeutic interventions to prevent or promote regression of severe COVID-19 may be an improvement of clinically relevant endpoints in biomodels that are as close as possible to the consequences of COVID-19. Clinical signs of COVID-19 range from mild to critical, with individuals with a mild form of the disease showing no signs, or mild pneumonia only. The severe disease is characterized by moderate to severe pneumonia. A critical form of COVID-19 includes the diagnosis of acute respiratory distress syndrome (ARDS), septic shock, and multiple organ failure. Risk factors, including older age, male gender, obesity, diabetes, and immunodeficiency, predispose people to the development of severe or critical forms of COVID-19 [1,2].

Histological analysis, surface visual inspection, and radiological imaging were the predominant approaches used to assess the development of inflammation and lung damage after experimental SARS-CoV-2 infection. However, these pathological observations have so far only been documented in some experimental animals. This is probably due to the focus of studies on early viral infection and its transmission. However, for rigorous drug efficacy studies, it is important that disease-relevant clinical signs can be measured in such a way that the effects of potential therapeutics on the consequences of COVID-19 can be statistically determined. This is why it is important to develop a relevant biomodel for monitoring the consequences of COVID-19. Previous studies for the in vivo monitoring of COVID-19 infection have been described in macaques [3], cats [4], ferrets [5], hamsters [6], mink [7], and transgenic mice that express human angiotensin I converting enzyme 2 (ACE2). These models have been used successfully for transmission and immunity studies, but only partially simulate the mechanisms involved in the consequences of COVID-19 [8]. The biggest limitation in the ACE2 transgenic mouse model is its lethal effects caused by neuroinvasion affecting the central nervous system [9,10].

In addition, many laboratories prefer to use a rat model for testing, which does not require the equipment of larger animals, and compared to mice, rat models are better adapted to simulate human diseases, apart from transgenic mice. Although there is not yet a relevant rat model for monitoring the consequences of severe COVID-19, there is a relatively good basis of biomodels, including spontaneously hypertensive, obese, diabetic, or immunodeficient rats, in which appropriate intervention can induce ARDS-like lung damage. The development of a rat model for monitoring the consequences of COVID-19 would bring new possibilities for monitoring drugs and substances that have the potential to treat COVID-19. Future studies are needed to standardize the relevant rat model of COVID-19 and protocols to allow comparisons of different drug candidate interventions.

We would like to invite all scientists and clinicians to send in their experimental and review articles devoted to the research of pathophysiological mechanisms of diseases, including COVID-19, on animal models to *Pathophysiology* [11], the flagship journal of the International Society for Pathophysiology (ISP) [12].
